# Response to Oxidative Stress Induced by Octahydro-1,3,5,7-tetranitro-1,3,5,7-tetrazocine in Differentiated PC12 Cells

**DOI:** 10.3390/toxics13050347

**Published:** 2025-04-27

**Authors:** Cunzhi Li, Xiaoqiang Lv, Zhiyong Liu, Hui Deng, Ting Gao, Huan Li, Xinying Peng, Airong Qian, Junhong Gao, Lifang Hu

**Affiliations:** 1 Laboratory for Bone Metabolism, Xi’an Key Laboratory of Special Medicine and Health Engineering, Key Laboratory for Space Biosciences and Biotechnology, Research Center for Special Medicine and Health Systems Engineering, School of Life Sciences, Northwestern Polytechnical University, No. 127 Youyi West Road, Beilin District, Xi’an 710072, China; 2 Toxicology Research Center, Institute for Hygiene of Ordnance Industry, No. 12 Zhangba East Road, Yanta District, Xi′an 710065, China; 3 Xi′an Key Laboratory of Toxicology and Biological Effect, Xi′an 710065, China

**Keywords:** HMX, PC12 cells, oxidative stress, free calcium ion, transcriptomics

## Abstract

Octahydro-1,3,5,7-tetranitro-1,3,5,7-tetrazocine (HMX) is a globally recognized energetic material that widely used in industrial, mining, and military fields. Like hexahydro-1,3,5-trinitro-1,3,5-triazine (RDX) and other nitramine compounds, HMX has also been reported to exhibit neurotoxicity. However, the molecular mechanisms underlying the toxic effects of HMX remain poorly understood. Therefore, this study aims to investigate the neurotoxicity induced by HMX by adopting PC12 cells. The results show that HMX treatment decreased cell viability and upregulated the intracellular free calcium ions (Ca^2+^) in PC12 cells. Furthermore, HMX caused aggravated oxidative stress in PC12 cells, as evidenced by the upregulations of reactive oxygen species (ROS) and malondialdehyde (MDA). Intracellular biochemical assays demonstrated that HMX induced loss of mitochondrial membrane potential in PC12 cells. Notably, altered expression of brain-derived neurotrophic factor (BDNF) and ionotropic glutamate receptors (iGluRs), as well as an abnormal transcription profile, were also observed in PC12 cells treated by HMX. These findings suggest that HMX exerts toxic effects on PC12 cells, involved in oxidative stress, and disturbances in Ca^2+^ and BDNF, accompanied by aberrant iGluRs. Overall, the present study helps us better understand the health hazards associated with HMX and provides valuable insights for developing the health protection standards related to HMX exposure.

## 1. Introduction

Energetic materials are substances with high energy density and chemical reactivity, widely used in military, aerospace, and mining fields [1]. Currently, energetic materials are regarded as serious threats to the health of workers, as most of them have toxicity and carcinogenic potential [2,3]. For example, TNT (2,4,6-trinitrotoluene) and RDX (cyclotrimethylenetrinitramine), which are also widely used worldwide, have caused health hazards to production workers and occupationally exposed military personnel [4,5,6]. Therefore, the toxicity of energetic materials has attracted increasing attention in society [7,8,9].

HMX (octahydro-1,3,5,7-tetranitro-1,3,5,7-tetrazocine, CAS#2691-41-0) is a widely used energetic material with excellent explosive performance. Nowadays, HMX is applied worldwide in industry, mining, aerospace, and military fields [10,11]. Acute toxicity studies demonstrated that the lethal dose 50 (LD_50_) for HMX is 2000 mg/kg for mice and 6500 mg/kg for rats [12]. Simultaneously, the LD_50_ values for acute oral exposure are close to 10,000 mg/kg in birds. In contrast, Cuthbert found that HMX is neurotoxic to New Zealand rabbits, with neurotoxic symptoms such as epilepsy, spasticity, and ataxia in rabbits exposed to HMX at 80 mg/kg orally [13]. The limits of exposure to HMX vary for different species, which may be related to their gastrointestinal absorption capacity. Toxicokinetic studies showed that 85% of the exposed dose of HMX was not absorbed in rats and was excreted [14]. Furthermore, seizures, nausea, convulsions, and loss of consciousness were observed in animals exposed to HMX [15]. HMX affects the liver and central nervous system and can accumulate in the kidneys, liver, brain, and heart, potentially leading to death in animals and histopathological damage in rodents [16]. In addition to model animals, HMX also caused hyperkinesia and seizures in lizards, symptoms of neurotoxicity, with an oral of 5 mg/kg·d [17]. These findings suggest that HMX is a neurotoxic substance. Nevertheless, the molecular mechanism underlying the neurotoxicity induced by HMX remain poorly understood.

PC12 is a cell line derived from rat adrenal medullary pheochromocytoma, which is commonly used in neuroscience research, encompassing studies on neurotoxicity, neuroprotection, neurosecretion, neuroinflammation, and synaptogenesis [18]. Undifferentiated PC12 cells are round suspended cells lacking neuron-like synapses, morphologically distinct from neurons [19]. Under the action of nerve growth factors (NGF), PC12 cells become differentiated cells, which are regarded as neuron cells with the characteristics of neurons, such as the growth of cell protrusions, the formation of synapse-like structures, and electrical excitability properties [20]. The differentiated PC12 cell is a classic cell model adopted for neurotoxicity research [21,22,23,24].

Therefore, the current study was conducted with the objective of investigating the neurotoxicity of HMX on differentiated PC12 cells. Our results suggested that HMX has multiple toxic effects on PC12 cells, including increased oxidative stress, elevated free calcium ions and disrupted mitochondrial membrane potential. Moreover, HMX caused aberrant expression levels of brain-derived neurotrophic factor (BDNF), and ionotropic glutamate receptors (iGluRs) as well as an abnormal transcription profile in differentiated PC12 cells. Overall, this study could improve our understanding of the potential molecular mechanisms underlying HMX induced neurological disorders and diseases.

## 2. Materials and Methods

### 2.1. Materials

HMX (CAS: 2691-41-0) with a purity greater than 99%, was obtained from Xi’an Modern Chemistry Research Institute (Xi’an, China). Fluo-4 AM was purchased from YEASEN (#40704ES72, Shanghai, China). JC-1 Mitochondrial Membrane Potential Assay Kit was provided by MedChemExpress (#HY-K0601, MCE, Shanghai, China). ROS (#ML926281), MDA (#ML077384-2), GSH (#ML531010-2) and SOD (#ML077379-2) Elisa Kit were obtained from Enzyme-linked Biotechnology (Shanghai, China). HRP-conjugated goat anti-Rabbit IgG was bought from Proteintech (#SA00001-2, Wuhan, China). Specific monoclonal antibodies to the AMPAR subunit glutamate receptor 1 (GluA1, #13185) and NMDA subunit glutamate receptor 2B (GluN2B, #4207) were purchased from Cell Signaling Technology (Danvers, MA, USA). Rabbit polyclonal antibody to brain-derived neurotrophic factor (BDNF) was provided by Abcam (#ab226483, Cambridge, UK). All other chemicals used were of analytical grade.

### 2.2. Cell Culture

The differentiated PC12 neural cells treated with NGF were obtained from Procell (Wuhan, China). The cells were cultivated in RPMI-1640 (#SH30255, HyClone, Logan, UT, USA) medium, supplemented with 10% fetal bovine serum (#25300054, Gibco, Grand Island, NY, USA) and 1% penicillin–streptomycin (MCE, China) at 37 °C in a humidified atmosphere with 5% CO_2_ and 95% air. After the cells grew to approximately 80% confluence, they were digested with 0.25% trypsin (#25200056, Gibco, USA), and the collected cells were inoculated into new dishes to continue culture.

### 2.3. Cell Counting Kit-8 Assay

The viability of PC12 cells treated with HMX was assessed by Cell Counting Kit-8, a highly sensitive reagent for cell proliferation and cytotoxicity based on WST-8 (CCK-8; MCE, Shanghai, China). Briefly, differentiated PC12 cells were grown in 96-well plates, incubated with different concentrations of HMX (0–200 μg/mL) at 37 °C for 24 h. Then, the CCK-8 solution was added to each well for 4 h. The measurement of the absorbance was performed by a spectrophotometer microplate reader (Enspire2300, PerkinElmer, Waltham, MA, USA) at 450 nm. The results were calculated by the formula: Cell viability (%) = [(A_450_ of treated group − A_450_ of medium)/(A_450_ of untreated group − A_450_ of medium)].

### 2.4. Mitochondrial Membrane Potential Assay

The evaluation of the mitochondrial membrane potential (MMP) was performed using the fluorescent dye JC-1. In brief, PC12 cells were seeded into 6-well plates with 1 × 10^6^ cells per well and incubated with different concentrations of HMX (0–100 μg/mL) at 37 °C for 24 h. Then, the cells were collected and incubated with fluorescent dye JC-1 solution (final concentration 2 μM) for 30 min. The red fluorescence represented JC-1 aggregations and the green fluorescence represented JC-1 monomers, and MMP is expressed as a JC-1 red/green ratio.

### 2.5. Measurements of Intracellular Free Calcium Ion (Ca^2+^)

The detection of free calcium ion was performed using Fluo-4 AM, a dye emitting fluorescence after binding with calcium ions. In brief, PC12 cells were seeded into 6-well plates with 1 × 10^6^ cells per well and incubated with different concentrations of HMX (0–100 μg/mL) at 37 °C for 24 h. Then, the cells were rinsed with Hank’s Balanced Salt Solution (HBSS) for three times. Next, The Fluo-4 AM solution (5 μM with HBSS) was added and incubated for 30 min. After washing with HBSS for twice, the cells were incubated with HBSS for 30 min, to ensure complete de-esterification of the dye within the cells. Finally, the cells were collected and the fluorescence intensity was measured with Flow Cytometer.

### 2.6. Western Blot

PC12 cells were seeded into a 100 mm cell dish. After treatment with HMX, the PC12 cells were lysed with Radio Immunoprecipitation Assay (RIPA), a rapid cell lysis buffer (#HY-K1001, MCE, Shanghai, China) containing protease and phosphatase inhibitor mixtures (#HY-K0013, MCE, Shanghai, China). Then, the protein concentration was analyzed using a BCA protein assay kit (#SK1070, Coolaber, Beijing, China) according to the manufacturer’s guide. Total proteins were heated with 5× loading buffer at 95 °C for 10 min to denature the proteins. Protein samples (20 μg total protein) were then separated in 10%–15% SDS-PAGE gels and transferred onto nitrocellulose membranes (#IPVH00010, Millipore, Bedford, MA, USA). After blocking with 5% milk at room temperature for 1 h, the membranes were incubated in primary antibodies against GluA1 (1:1000), GluN2B (1:1000) and BDNF (1:1000) overnight. Then, they were rinsed with PBST for 25 min, and the membranes were incubated with HRP-conjugated secondary antibody (1:2000) for 1 h at room temperature. Finally, the protein bands in membranes were visualized by ECL reagent (#WBKLS0100, Millipore, USA). The expression of all proteins was normalized to GAPDH in the corresponding membranes.

### 2.7. Enzyme-Linked Immunosorbent Assay (ELISA)

The ELISA protocol is performed according to the manufacturer’s instructions. Briefly, after treatment with HMX, the cells were lysed with RIPA. Then, the standard sample and the cell fraction to be tested were added to the assay 96-well plate, followed by incubation at 37 °C for 30 min. Next, HRP or biotinylated detection antibodies and the chromogenic substrate TMB were added to the plate. Finally, the reaction was stopped by adding H_2_SO_4_, and the optical density (OD) was measured at 450 nm using a microplate reader (Enspire2300, PerkinElmer). The level of the target product is related to the OD, which is calculated after a four-parameter logistic transformation.

### 2.8. RNA Sequencing

Transcriptome sequencing in this study was based on the Illumina sequencing platform to investigate all mRNAs transcribed from specific PC12 cells after treatment with HMX. Reference genome version: ensembl_111_rattus_norvegicus_mratbn7_2_toplevel.

The RNA-seq workflow consists of two main parts: library preparation and bioinformatics analysis. Initially, extracted RNA was precisely tested for integrity using an Agilent 2100 bioanalyzer (Agilent, Palo Alto, CA, USA). Poly-T oligo-attached magnetic beads were used to bind specifically to the poly(A) tail of the mRNA to remove other RNAs. Subsequently, the first-strand cDNA was synthesized using the M-MuLV reverse transcriptase system, and the second-strand cDNA was synthesized in the DNA polymerase I system. Then, cDNA fragments of 370–420 bp in length were amplified by PCR and purified again using AMPure XP beads to establish the library. After the libraries were qualified for identification, they were subjected to Illumina sequencing. Briefly, fluorescently labeled dNTPs, DNA polymerases, and other reaction materials were used in the PCR reaction system, and the fluorescence signal was converted into sequencing peaks to obtain the sequencing information of the fragments.

Low-quality data and sequences containing poly-N were removed from the raw data (FASTQ format) to obtain clean data. HISAT2 v2.0.5 was then used to align the clean data to the reference genome. In featureCounts v1.5.0-p3 software, the FPKM of each gene was calculated to determine the expression level of the gene. Differential expression analysis of the two groups was performed using DESeq2 R package (1.20.0), and genes with an adjusted *p*-value ≤ 0.05 were defined as differentially expressed. Next, Gene Ontology (GO) enrichment analysis of differentially expressed genes was performed using the clusterProfiler (3.8.1) software, and GO terms with an adjusted *p*-value ≤ 0.05 were significantly enriched by the differentially expressed genes. KEGG is a database that integrates genomic, chemical, and systems functional information. The enrichment of differentially expressed genes in KEGG pathways was analyzed using the clusterProfiler (3.8.1) software.

### 2.9. Statistical Analysis

Experiments were performed in triplicate and repeated three times with similar results. GraphPad Prism 8 (GraphPad, La Jolla, CA, USA) was used for statistical analysis. Significant differences were evaluated by One-way ANOVA. All data were presented as mean ± standard error of the mean (SEM), and values of *p* less than 0.05 were considered significant.

## 3. Results

### 3.1. HMX Induced Cytotoxicity in Differentiated PC12 Cells

**Figure 1:**
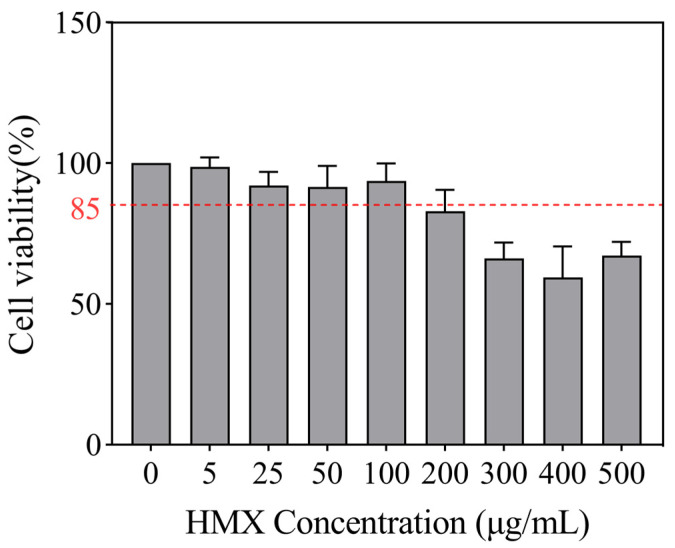
Effects of HMX on cell viability in differentiated PC12 cells. After 24 h treatment of different concentrations (5 to 500 μg/mL) of HMX, the cell viability of differentiated PC12 cells was detected by CCK-8 assay. The red dashed line indicates cell viability at 85%, *n* = 3.

Cell viability reflects the health status of cells and is an important indicator for assessing the cytotoxicity of substances. The CCK-8 assay was used to analyze the cytotoxic effects of HMX on PC12 cells. A concentration-dependent decrease in cell viability was observed in HMX-treated PC12 cells (Figure 1). HMX at concentrations 5 to 100 μg/mL did not induce cytotoxicity, with cell viability remaining above 85%. Therefore, non-cytotoxic concentrations of HMX ranging from 5 to 100 μg/mL were chosen for PC12 cell exposure.

### 3.2. HMX Increased Oxidative Stress in Differentiated PC12 Cells

**Figure 2:**
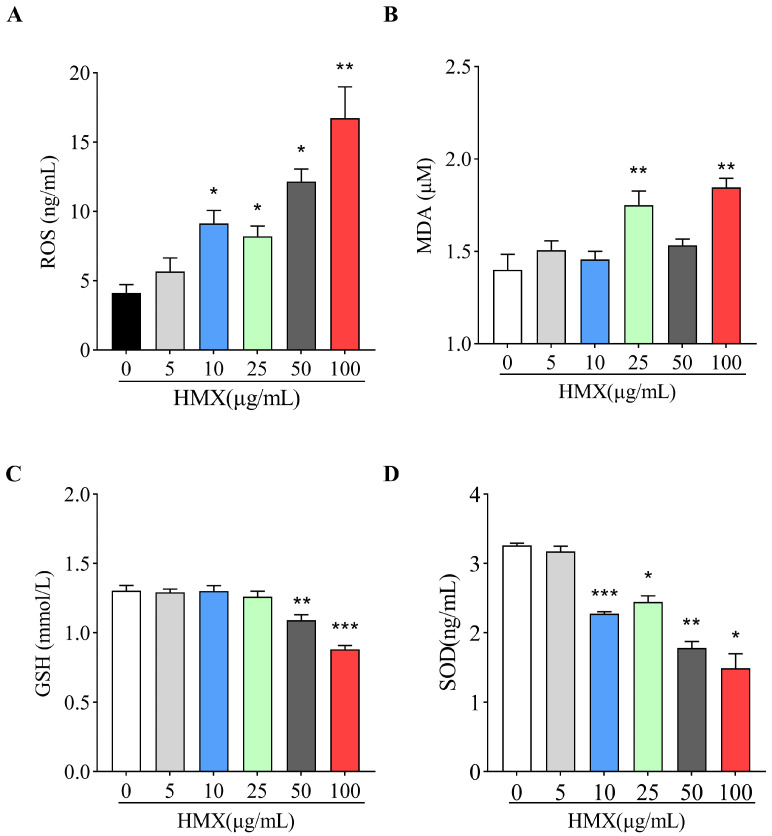
HMX exposure leads to oxidative stress in differentiated PC12 cells. Effects of 0–100 μg/mL HMX for 24 h on (**A**) reactive oxygen species (ROS), (**B**) malondialdehyde (MDA), (**C**) glutathione (GSH), and (**D**) superoxide dismutase (SOD) in differentiated PC12 cells. All data are presented as mean ± SEM. * *p* < 0.05, ** *p* < 0.01, *** *p* < 0.001, *n* = 3, one-way ANOVA.

Oxidative stress generally refers to the imbalance between the oxidative and antioxidant systems caused by excessive ROS in cells or tissues. Since neurons are extremely susceptible to oxidative stress, we examined the effects of HMX on oxidants (ROS and MDA) and antioxidants (SOD and GSH) in PC12 cells. The ELISA detection showed that exposure of PC12 cells to HMX (10, 25, 50 and 100 μg/mL) caused excessive production of oxidative ROS (Figure 2A). Meanwhile, a significant increase in MDA was found in PC12 cells treated by 25 and 100 μg/mL of HMX (Figure 2B). However, the levels of antioxidants GSH were significantly decreased in PC12 cells treated by 50 and 100 μg/mL of HMX (Figure 2C). In PC12 cells treated by 10–100 μg/mL of HMX, the amount of MDA was significantly decreased (Figure 2D). Taken together, these results indicated that HMX induced excessive oxidative stress in PC12 cells.

### 3.3. HMX Resulted in Lower Mitochondrial Membrane Potential (MMP) in PC12 Cells

**Figure 3:**
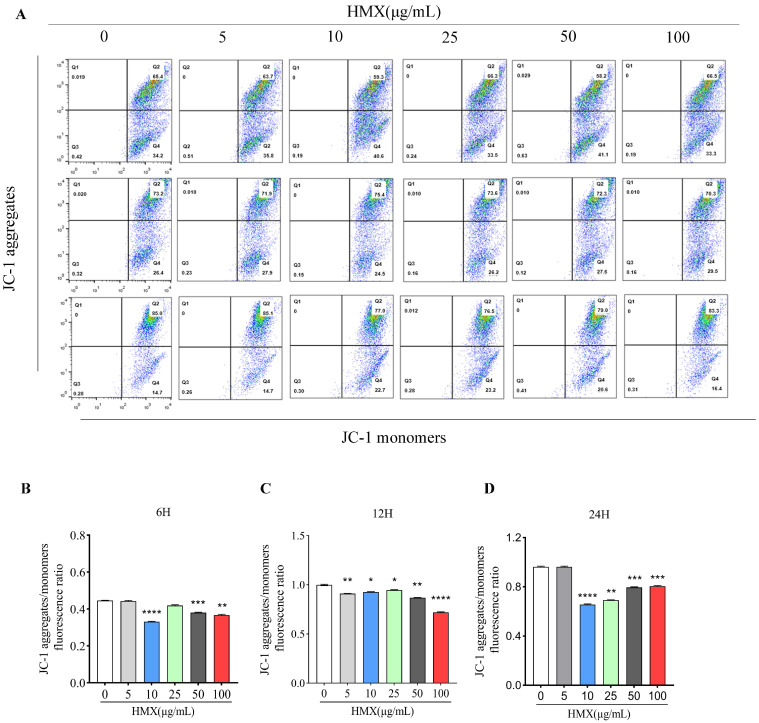
A decrease in mitochondrial transmembrane potential in differentiated PC 12 cells treated by HMX. Mitochondrial membrane potential measured by flow cytometry of JC-1 in PC12 cells treated by HMX at 5 to 100 μg/mL. (**A**) representative images of flow cytometry of JC-1. JC-1 aggregates/monomers fluorescence ratio in HMX-treated PC12 cells for 6 h (**B**), 12 h (**C**) and 24 h (**D**). All data are presented as mean ± SEM. * *p* < 0.05, ** *p* < 0.01, *** *p* < 0.001, **** *p* < 0.0001, *n* = 3, one-way ANOVA.

Mitochondria is the main organelle of ROS production, and excess ROS results in a decrease in mitochondrial membrane potential [25,26]. Decreased MMP is a hallmark event in the stages of apoptosis in damaged cells. For neurons, mitochondrial energy metabolism disorders can cause abnormal neuronal structure and function and induce neurotoxicity [27,28]. The result of JC-1 flow cytometry indicated that treatment with HMX for 6, 12, and 24 h disrupted the MMP in PC12 cells (Figure 3A). As is shown in Figure 3B, compared with the control group (0 μg/mL), MMP in cells treated with HMX for 6 h (10, 50, and 100 μg/mL) was significantly decreased. Furthermore, in the 12 h treatment, MMP in all HMX-treated cells exhibited dose-dependent reductions (Figure 3C). In addition, in the cells treated with 10, 25, 50, and 100 μg/mL of HMX for 24 h, MMP was significantly decreased compared with the control group (Figure 3D). This result indicated that HMX caused a disruption in MMP in PC12 cells.

### 3.4. HMX Increased Free Calcium Ion (Ca^2+^) in Differentiated PC12 Cells

**Figure 4:**
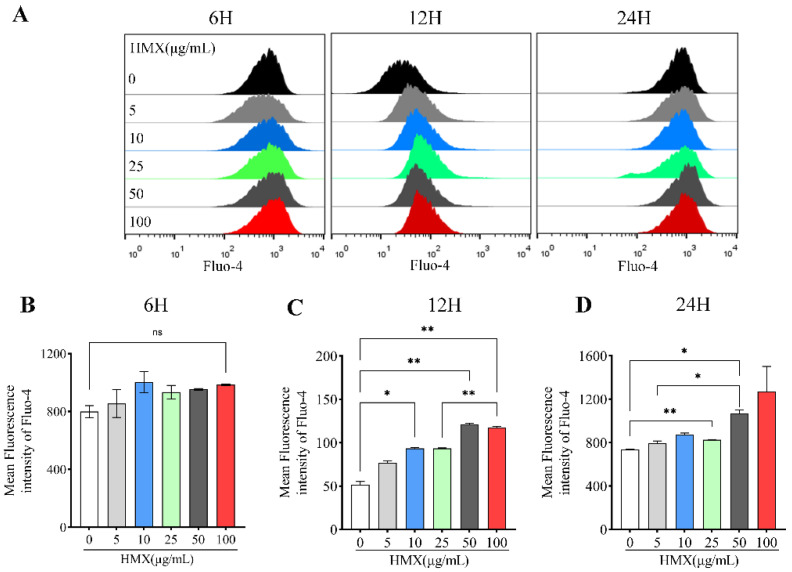
HMX treatment resulted in increased calcium levels in differentiated PC12 cells. Concentration of free calcium ion (Ca^2+^) measured by flow cytometry of Fluo-4AM in PC12 cells treated by HMX at 5 to 100 μg/mL. (**A**) representative images of flow cytometry of Fluo-4AM. Mean fluorescence intensity of Fluo-4AM in HMX-treated PC12 cells for 6 h (**B**), 12 h (**C**) and 24 h (**D**). All data are presented as mean ± SEM. * *p* < 0.05, ** *p* < 0.01, ns, no significant difference, *n* = 3 per groups, one-way ANOVA.

Ca^2+^ is one of the important signaling molecules for mitochondrial membrane potential regulation, and the concentration of Ca^2+^ is correlated with mitochondrial membrane potential [29,30]. Calcium plays a key role in the nervous system, which is essential for the regulation of neuronal plasticity and accurate nerve signal transduction [31]. The dysregulation of calcium homeostasis damages mitochondria and inhibits cellular respiration, leading to a decrease in ATP and ultimately neuronal death [32,33]. In order to evaluate the changes in free calcium ion induced by HMX, we performed flow cytometry to assess fluorescence intensity of Fluo-4 AM, a specific calcium probe. PC12 cells were treated with HMX for 6, 12, and 24 h. The results showed that HMX led to an increased concentration of intracellular calcium ions in PC12 cells (Figure 4A). For 6 h treatment, there was no significant difference between the control group and HMX-treated PC12 cells in the fluorescence intensity of Fluo-4 AM (Figure 4B). However, in the 12 h treatment, HMX increased the fluorescence intensity of Fluo-4 AM, accompanied by a clear dose-effect relationship (Figure 4C). Consistent with 12 h, in the cells treated with HMX, fluorescence intensity of Fluo-4 AM was significantly increased at 24 h (Figure 4D). Collectively, these data demonstrated that HMX induced an increased concentration of Ca^2+^ in PC12 cells.

### 3.5. HMX Reduced BDNF Expression but Increased GluN2B Expression in Differentiated PC12 Cells

**Figure 5:**
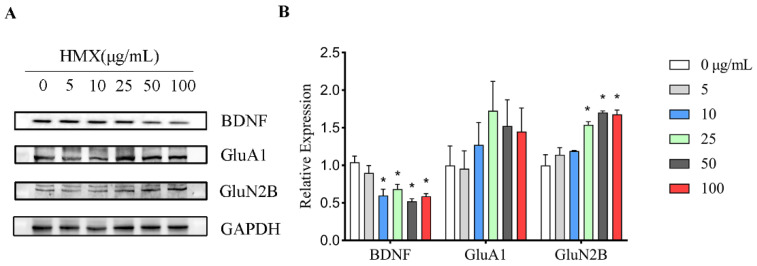
Reduced expression of BDNF and higher expression of GluA1in differentiated PC12 cells treated by HMX. Western Blot was performed to evaluate the expressions of BDNF, GluA1 and GluN2B protein in PC12 cells treated with HMX for 24 h. (**A**) Representative images of Western blot of BDNF, GluA1 and GluN2B in differentiated PC12 cells treated by HMX at 5 to 100 μg/mL. (**B**) Quantification of Western blot in (**A**). All data are presented as mean ± SEM. * *p* < 0.05, indicates significant differences between the exposure control and HMX groups, *n* = 3, one-way ANOVA.

Highly differentiated PC12 cells express brain-derived neurotrophic factor (BDNF) and glutamate receptors. Aberrant expression levels of BDNF and glutamate receptors have been reported to result in excitatory neurotoxicity, so the expression levels of these proteins in PC12 cells were analyzed by Western blot (Figure 5A). We found a reduction in the expression of BDNF in HMX treated PC12 cells. Furthermore, we observed an interesting increase in the expression of GluN2B (Figure 5B). This result suggested that HMX exposure alters proteins associated with the nervous system in PC12 cells.

### 3.6. HMX Caused Abnormal Transcription Profile in Differentiated PC12 Cells

**Figure 6:**
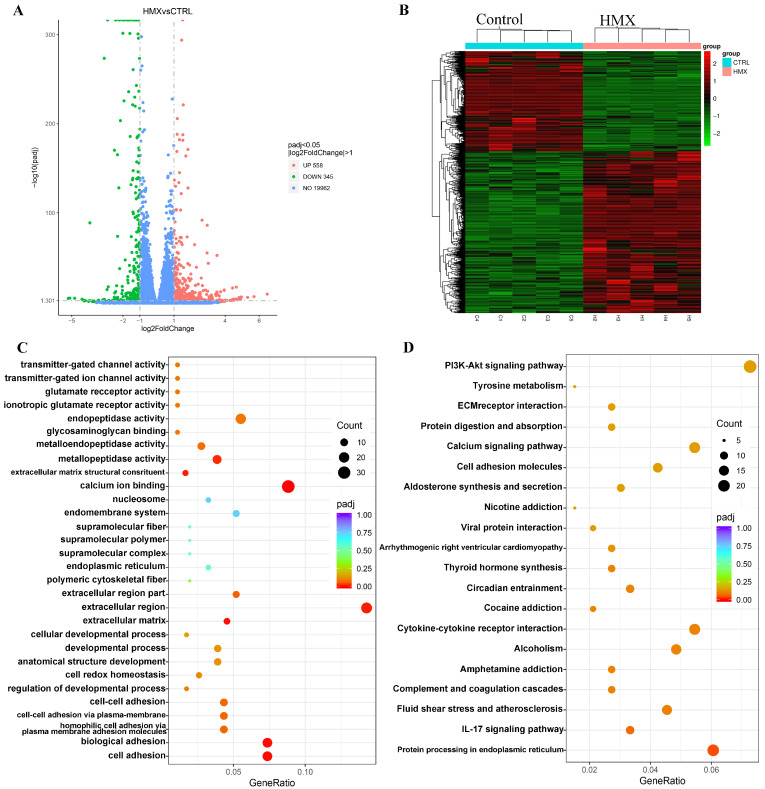
HMX caused significant changes in the transcription profile of differentiated PC12 cells. (**A**) Volcano plot of the differentially expression genes (DEGs) in PC12 cells. (**B**) Heat map of the DEGs. (**C**) Gene ontology (GO) analysis of DEGs in PC12 cells. The area of circles indicate number of DEGs and the color of circles represents adjusted P values between control group and HMX group. (**D**) KEGG pathway (top 20) analysis of DEGs in PC12 cells.

To further uncover the potential molecular targets and possible mechanisms underlying the multiple neurotoxic effects of HMX on differentiated PC12 cells, we performed transcriptome sequencing on differentiated PC12 cells treated with HMX. PC12 cells were exposed to HMX at a concentration of 50 μg/mL for 24 h, followed by transcriptomic analysis. As shown in the volcano plot, a total of 20,863 genes were identified, and 903 genes were identified as differentially expressed genes (DEGs), of which 558 genes were significantly upregulated and 345 were significantly downregulated (Figure 6A). A heatmap was generated to show the expression levels of DEGs between the control group and the HMX-treated group (Figure 6B). Next, the Kyoto Encyclopedia of Genes and Genomes (KEGG) pathway analysis of the DEGs was performed, and the twenty most significantly affected pathways are shown in Figure 6C,D. In the Enriched GO pathways, a lot of differentially expressed genes were enriched on calcium ion binding, ionotropic glutamate receptor activity, and glutamate receptor activity, which play key roles in CNS (Table 1). In the result of Western blot, we found that HMX treatment caused an alteration in the expression of AMPAR-type receptor GluA1 in PC12 cells. Furthermore, *Gria2*, *Grin3b*, *Grin2d* and *Grin1* were also abnormally regulated, similar to GluA1, encoding ionotropic glutamate receptors (AMPAR or NMDAR), which are essential for the proper transmission of neural signals. Additionally, we noted that in the KEGG pathways, many differentially expressed genes were enriched in the calcium signaling pathway and the PI3K-Akt signaling pathway (Table 2). *Calm2* and *Calml4* encode calmodulin proteins, which interacts with calcium, and activated calmodulin has been shown to regulate the expression of BDNF. PI3K-Akt signaling pathway is involved in a variety of physiological processes, which can be activated by oxidative stress, and PI3K/AKT signaling can in turn positively regulate ROS production [34].

Taken together, some of the more noticeable changes in nervous system-related GO and KEGG pathways include calcium ion binding, calcium signaling pathway and glutamate receptor activity. Taken together, the results of RNA sequencing indicated that HMX induced significant changes in the transcription profile of PC12 cells, and the enrichments of DEGs revealed possible pathways and molecular mechanisms involved in HMX-induced neurotoxicity in differentiated PC12 cells.

## 4. Discussion

HMX is widely used in the manufacturing of explosives and propellants, commonly found in mining, military, and aerospace fields [35]. Several studies have reported neurological effects in animals after acute exposure to HMX; however little is known about the underlying mechanism of the neurotoxicity induced by HMX [36]. Here, we investigated the toxic effects of HMX on highly differentiated PC12 cells, a classic cell model adopted for neurotoxicity research. We showed that HMX increased oxidative stress and Ca^2+^, accompanied by lower mitochondrial membrane potential and abnormal transcription profile in PC12 cells.

Oxidative stress (OS) refers to the excessive production of highly reactive molecules, such as reactive oxygen species (ROS), and an imbalance between oxidative and antioxidant systems in the cells, which leads to cytotoxic effects [37]. For nerve cells, the generation and elimination of ROS are generally in a state of dynamic balance and maintain the normal function of the cell [38]. We found in the present study that PC12 cells exposed to HMX had a redox imbalance and abnormally elevated ROS levels, suggesting that the cells were compromised by oxidative stress. First, the levels of oxidative substances, ROS and MDA were significantly increased in PC12 cells by treatment of HMX. MDA is an end product of lipid peroxidation, and the production of free radicals, such as ROS, which can lead to excess MDA, damaging mitochondria and cell membranes [39]. In addition, the levels of both antioxidant substances decreased to varying extents. GSH (glutathione) is an important antioxidant with some detoxification functions, and present in most cells [40]. Superoxide dismutase (SOD) is an antioxidant metalloenzyme present in organisms and plays a crucial role in maintaining the balance between oxidation and anti-oxidation in the body [41,42]. Nerve cells have been shown to be highly sensitive to oxidative stress, given that a significant portion of the energy required by neurons is supplied by mitochondria [43]. In the mitochondrial membrane potential assay using JC-1, we observed an abnormal decline in mitochondrial membrane potential in PC12 cells exposed to HMX. Abnormal mitochondrial membrane potential is likely to result in the dysfunction of the mitochondrial respiratory chain [44]. In neurons, one of the primary sources of ROS is the physiological processes such as mitochondrial respiratory chain dysfunction. Research has demonstrated that neural cells are particularly vulnerable to oxidative stress caused by excessive ROS [45,46].

Therefore, we concluded that HMX exposure leads to excessive oxidative stress and compromises the mitochondrial membrane potential in PC12 cells. In addition, oxidative stress has been demonstrated to play a critical role in the neurotoxicity caused by various compounds, such as mercury, cadmium, and aluminum [47,48,49,50]. Our previous study showed that CL-20, another nitrosamine compound, induced persistent stress, leading to mutations in mitochondrial genes and DNA damage in V79 cells [51]. Additionally, ROS-induced oxidative stress is also observed in the ancient nitroamine energy-containing compound 2,4,6-trinitrotoluene (TNT) [52].

Calcium ions (Ca^2+^) are critical for the optimal functioning and survival of neurons [53]. As reported, excess of Ca^2+^ can lead to excitotoxicity in neurons, triggering downstream signaling pathways that activate various deleterious enzymes and result in mitochondrial damage [54]. Specifically, elevated levels of Ca^2+^ can active membrane phosphatidic acid, initiating a cascade of event that culminate in lipid peroxidation and the production of free radicals [55]. Moreover, increased intracellular calcium concentrations contribute to the deposition of calcium within mitochondria, damaging mitochondrial membrane potential [56,57]. In neurons, calcium interacts with calmodulin (CaM), and activated calmodulin has been shown to regulate the expression of BDNF [58,59,60]. In line with this, transcriptome data indicate that *Calm 2* and *Calm 4* are differentially expressed genes in PC12 cells treated with HMX. Additionally, previous findings have demonstrated that in some instances of excitotoxic injury, the expression of neurotrophic factors such as BDNF is significantly diminished in brain tissue [61], which is consistent with the observed reduction in BDNF expression in differentiated PC12 cells treated with HMX.

An analysis of transcriptome sequencing data revealed that many genes in the Mmp family (*Mmp3*, *Mmp9*, *Mmp10*, *Mmp11*, *Mmp13*, *Mmp15* and *Mmp19*) were enriched into multiple pathways, such as extracellular region, extracellular region part, metalloendopeptidase activity and endopeptidase activity. Among the altered *Mmp*s, MMP-9 is also proposed as an Alzheimer’s marker. Neuronal MMP-9 participates in synaptic plasticity by controlling the shape of dendritic spines and the function of excitatory synapses, and if not released properly, MMP-9 can cause a variety of brain diseases, including epilepsy, schizophrenia, and neurodegeneration [62].

In addition, we found a number of Cdh family and Pcdh family genes associated with calcium ion adhesion that mediate specific cell–cell adhesion in a calcium-dependent manner, allowing cells to interact with the environment [63]. As reported, *Cdh5*, *Cdh16*, *Cdh26*, and *Pcdhb10* are enriched into cell adhesion and calcium ion binding signaling pathways.

The differentially expressed genes *Gria2*, *Grin3b*, *Grin2d* and *Grin1* were enriched into ionotropic glutamate receptor activity, glutamate receptor activity, transmitter-gated ion channel activity and neurotransmitter receptor activity pathways. The *Gria2* gene encodes the GluR2 subunit of the AMPA receptor, polymorphisms of which are associated with psychiatric disorders such as schizophrenia, major depressive disorder, and bipolar disorder [64]. The Grin family genes encode the glutamate ionotype receptor NMDA type, which has been shown to be involved in neurological disorders such as neurodevelopmental disorders. Consistent with our findings, the previous WB experiments also indicated differentially expressed GluA1 protein, although the NMDA isoforms were different.

Additionally, we found that many differentially expressed genes were enriched on the calcium signaling pathway, such as *Atp2b4*, *Atp2a3*, *Flt1*, *Fgf22*, *Plcd4*, *Nos3*, *Itpka*, *Grin2d*, *Cacna1h*, *Ptk2b*, *Grin1*, *Calm2*, *Calml4*, *Fgfr4*, *Fgf21*, *Plcb2*, *Adra1d* and *Ntrk3*. For example, the *Fgf22* gene plays an irreplaceable role in brain development and the formation, stability and regulation of central nervous synapses [65]. These calcium pathway-related genes play important functions in the nervous system by regulating intracellular calcium signaling.

In summary, we believed that HMX-induced abnormal calcium signal in PC12 cells may be the main reason for its toxic effects. On the one hand, excess calcium can result in mitochondrial damage and broken mitochondrial membrane potential, causing the dysregulation of mitochondrial function. Abnormalities in mitochondrial membrane potential and function lead to an imbalance in the oxidative and reduction systems, resulting in excess ROS and SOD within cells. On the other hand, aberrant calcium signaling induced abnormalities in a large number of calcium-related gens, such as *Cdh*, *Pcdh*, *Mmp*, *Atp2b* and *Calm*, among which *Calm* has an important regulatory effect on neurotrophic factors such as BDNF. In addition, the neuronal ionic glutamate receptors *Grin* and *Gria*, which are calcium-permeable ion channels, also showed aberrant expressions. Taken together, calcium-related homeostasis dysregulation and abnormal calcium signaling may be important reasons for many toxic effects of HMX in PC12 cells.

In conclusion, the findings of our study demonstrated that HMX induced cytotoxicity in differentiated PC12 cell lines, accompanied by oxidative stress, disrupted mitochondrial membrane potential, disturbance of Ca^2+^ and changes in expression of BDNF. Overall, the findings from this study may provide clues to uncover the neurotoxicity of HMX, helping to develop protective measures and effective therapeutic strategies during the production process of HMX.

## 5. Conclusions

In conclusion, our findings indicated that HMX exerts multiple toxic effects on high-differentiated PC12 cells, including excessive oxidative stress and Ca^2+^, aberrant BDNF and iGluRs, accompanied by abnormal transcription profile.

## Figures and Tables

**Table 1:** GO pathways and involved gene name.

GO Pathway	Gene Name
Cell adhesion	*Lama4*/*Lama2*/*Ncan*/*Thbs2*/*Cdh5*/*Hapln1*/*Sned1*/*Pcdhb10*/*Hapln2*/*LOC108348201*/*Cdh16*/*Dsc3*/*Fat2*/*Pcdhb7*/*Pcdhb11*/*Fat3*/*Cdh26*
Biological adhesion	*Lama4*/*Lama2*/*Ncan*/*Thbs2*/*Cdh5*/*Hapln1*/*Sned1*/*Pcdhb10*/*Hapln2*/*LOC108348201*/*Cdh16*/*Dsc3*/*Fat2*/*Pcdhb7*/*Pcdhb11*/*Fat3*/*Cdh26*
Homophilic cell adhesion via plasma membrane adhesion molecules	*Cdh5*/*Pcdhb10*/*LOC108348201*/*Cdh16*/*Dsc3*/*Fat2*/*Pcdhb7*/*Pcdhb11*/*Fat3*/*Cdh26*
Cell–cell adhesion via plasma-membrane adhesion molecules	*Cdh5*/*Pcdhb10*/*LOC108348201*/*Cdh16*/*Dsc3*/*Fat2*/*Pcdhb7*/*Pcdhb11*/*Fat3*/*Cdh26*
Cell–cell adhesion	*Cdh5*/*Pcdhb10*/*LOC108348201*/*Cdh16*/*Dsc3*/*Fat2*/*Pcdhb7*/*Pcdhb11*/*Fat3*/*Cdh26*
Regulation of developmental process	*Lama4*/*Lama2*/*Palmd*/*Csf3*
Cell redox homeostasis	*Pdia6*/*Pdia3*/*Pdia4*/*Txndc11*/*Glrx*/*Pdia5*
Anatomical structure development	*Lama4*/*Lama2*/*Spry1*/*Palmd*/*Siah3*/*Csf3*/*Draxin*/*Prm2*/*Crx*
Developmental process	*Lama4*/*Lama2*/*Spry1*/*Palmd*/*Siah3*/*Csf3*/*Draxin*/*Prm2*/*Crx*
Cellular developmental process	*Palmd*/*Csf3*/*Draxin*/*Prm2*
Extracellular matrix	*Mmp19*/*Mmp15*/*Mmp11*/*Mmp3*/*Mmp10*/*Mmp13*/*Mmp9*
Extracellular region	*Mmp19*/*Stc2*/*Mmp15*/*Ccl2*/*Igfbp7*/*Mmp11*/*Nppb*/*Mmp3*/*Plat*/*Mmp10*/*Thbs2*/*Pinlyp*/*Umodl1*/*Mmp13*/*Mmp9*/*Csf2*/*Esm1*/*Draxin*/*Igfbp3*/*C3*/*Htra3*/*Chit1*
Extracellular region part	*Mmp19*/*Mmp15*/*Mmp11*/*Mmp3*/*Mmp10*/*Mmp13*/*Mmp9*/*C3*
Polymeric cytoskeletal fiber	*Des*/*Nefm*/*LOC102555739*
Endoplasmic reticulum	*Calr*/*Canx*/*P4ha1*/*Selenos*/*Calr4*
Supramolecular complex	*Des*/*Nefm*/*LOC102555739*
Supramolecular polymer	*Des*/*Nefm*/*LOC102555739*
Supramolecular fiber	*Des*/*Nefm*/*LOC102555739*
Endomembrane system	*Calr*/*Canx*/*P4ha1*/*Uso1*/*Csgalnact2*/*Selenos*/*Calr4*/*Spesp1*
Nucleosome	*Hist3h2ba*/*H2ac25*/*Hist1h3b*/*ENSRNOG00000064540*/*H2bc18*
Calcium ion binding	*Calr*/*Canx*/*Creld2*/*LOC100910088*/*Nucb2*/*Sparc*/*Ltbp4*/*Notch4*/*Efhd1*/*Lcp1*/*Thbs2*/*Ppef1*/*Umodl1*/*Padi3*/*Cdh5*/*Hmcn2*/*Pcdhb10*/*Calm2*/*LOC108348201*/*Calml4*/*Cdh16*/*Calr4*/*Tescl*/*Plcb2*/*Dsc3*/*Fat2*/*Pcdhb7*/*Pcdhb11*/*RGD1559821*/*Fat3*/*Cdh26*
Extracellular matrix structural constituent	*Col5a2*/*Col5a3*/*Col3a1*/*Col1a2*/*Col4a3*/*Col11a1*
Metallopeptidase activity	*Mmp19*/*Mmp15*/*Mmp11*/*Adamts4*/*Mmp3*/*Mmp10*/*Cpm*/*Mmp13*/*Mmp9*/*Ace*/*Aebp1*/*Adam11*/*Adamts5*/*Cpz*
Metalloendopeptidase activity	*Mmp19*/*Mmp15*/*Mmp11*/*Adamts4*/*Mmp3*/*Mmp10*/*Mmp13*/*Mmp9*/*Adam11*/*Adamts5*
Glycosaminoglycan binding	*App*/*Ncan*/*Hapln1*/*Hapln2*
Endopeptidase activity	*Mmp19*/*Mmp15*/*Mmp11*/*Prss22*/*Adamts4*/*Mmp3*/*Plat*/*Masp1*/*Mmp10*/*Mmp13*/*Ctrb1*/*Pcsk2*/*Mmp9*/*RGD1559662*/*Adam11*/*Klk13*/*Rhbdl2*/*Adamts5*/*Cym*/*Plg*
Ionotropic glutamate receptor activity	*Gria2*/*Grin3b*/*Grin2d*/*Grin1*
Glutamate receptor activity	*Gria2*/*Grin3b*/*Grin2d*/*Grin1*
Transmitter-gated ion channel activity	*Gria2*/*Grin3b*/*Grin2d*/*Grin1*
Transmitter-gated channel activity	*Gria2*/*Grin3b*/*Grin2d*/*Grin1*
Neurotransmitter receptor activity	*Gria2*/*Grin3b*/*Grin2d*/*Grin1*

**Table 2:** KEGG pathways and involved gene name.

KEGG Pathway	Gene Name
Protein processing in endoplasmic reticulum	*Hspa5*/*Hsp90b1*/*Calr*/*Pdia6*/*Canx*/*Sel1l*/*Pdia3*/*Pdia4*/*Hyou1*/*Herpud1*/*Dnajc3*/*Ero1b*/*Ddit3*/*Dnajb11*/*Atf6*/*Xbp1*/*Syvn1*/*Selenos*/*Casp12*/*Calr4*
IL-17 signaling pathway	*Hsp90b1*/*Ccl2*/*Ccl7*/*Cxcl10*/*Mmp3*/*Fosb*/*Lcn2*/*Mmp13*/*Mmp9*/*Csf2*/*Csf3*
Fluid shear stress and atherosclerosis	*Hsp90b1*/*Sqstm1*/*Ccl2*/*Plat*/*Map2k6*/*Cdh5*/*Nos3*/*Gstm7*/*Mmp9*/*Calm2*/*Calml4*/*Ncf2*/*Itgb3*/*Gstt3*
Complement and coagulation cascades	*F3*/*Plat*/*Masp1*/*Itgax*/*Vwf*/*F13a1*/*C3*/*Plg*/*C1qc*
Amphetamine addiction	*Creb3l1*/*Gria2*/*Fosb*/*Grin3b*/*Th*/*Grin2d*/*Grin1*/*Calm2*/*Calml4*
Alcoholism	*Creb3l1*/*Fosb*/*Grin3b*/*Gng3*/*Th*/*Grin2d*/*Hist3h2ba*/*Grin1*/*Calml2*/*Calml4*/*LOC102549173*/*AC130391.1*/*Hist1h3b*/*ENSRNOG00000064540*/*H2bc18*
Cytokine–cytokine receptor interaction	*Ccl2*/*Ccl7*/*Bmp7*/*Cxcl10*/*Tnfrsf1b*/*Il33*/*Eda2r*/*Tnfsf18*/*Ccr7*/*Csf2*/*Il5ra*/*Tnfrsf9*/*Csf3*/*Il11ra1*/*Tslp*/*Ccl27*
Cocaine addiction	*Creb3l1*/*Gria2*/*Fosb*/*Grin3b*/*Th*/*Grin2d*/*Grin1*
Circadian entrainment	*Gria2*/*Gng3*/*Adcyap1r1*/*Grin2d*/*Cacna1h*/*Rasd1*/*Grin1*/*Calm2*/*Calml4*/*Plcb2*
Thyroid hormone synthesis	*Hspa5*/*Hsp90b1*/*Canx*/*Pdia4*/*Creb3l1*/*Gpx1*/*Atp1a2*/*Tg*/*Plcb2*
Arrhythmogenic right ventricular cardiomyopathy	*Lama2*/*Atp2a3*/*Sgcd*/*Des*/*Cacng8*/*Dsp*/*Itgb7*/*Itgb3*/*Sgcg*
Viral protein interaction with cytokine and cytokine receptor	*Ccl2*/*Ccl7*/*Cxcl10*/*Tnfrsf1b*/*Ccr7*/*Ccl27*
Nicotine addiction	*Gria2*/*Grin3b*/*Slc17a6*/*Grin2d*/*Grin1*
Aldosterone synthesis and secretion	*Creb3l1*/*Atp2b4*/*Atp1a2*/*Star*/*Pde2a*/*Cyp11a1*/*Cacna1h*/*Calm2*/*Calml4*/*Plcb2*
Cell adhesion molecules	*Sdc3*/*Alcam*/*Siglec1*/*Cldn4*/*Cdh5*/*Spn*/*Cd80*/*Cntn2*/*Ptprf*/*RT1-M6-2*/*Itgb7*/*Esam*/*Nectin1*/*Jam2*
Calcium signaling pathway	*Atp2b4*/*Atp2a3*/*Flt1*/*Fgf22*/*Plcd4*/*Nos3*/*Itpka*/*Grin2d*/*Cacna1h*/*Ptk2b*/*Grin1*/*Calm2*/*Calml4*/*Fgfr4*/*Fgf21*/*Plcb2*/*Adra1d*/*Ntrk3*
Protein digestion and absorption	*Col5a2*/*Col5a3*/*Col12a1*/*Col14a1*/*Atp1a2*/*Ctrb1*/*Col9a3*/*Col3a1*/*Col11a1*
ECM–receptor interaction	*Thbs1*/*Lama4*/*Lama2*/*Lamb2*/*Thbs2*/*Col9a3*/*Itgb7*/*Vwf*/*Itgb3*
Tyrosine metabolism	*Aox1*/*Aox2*/*Th*/*Adh6*/*RGD1308564*
PI3K-Akt signaling pathway	*Hsp90b1*/*Thbs1*/*Lama4*/*Lpar1*/*Creb3l1*/*Lama2*/*Lamb2*/*Pck2*/*Flt1*/*Thbs2*/*Fgf22*/*Areg*/*Nos3*/*Col9a3*/*Gng3*/*Csf3*/*Itgb7*/*Fgfr4*/*Tgfa*/*Fgf21*/*Vwf*/*Itgb3*/*Gys2*

## Data Availability

All data included in this study are available upon request via contact with the corresponding author (Junhong Gao, gaoxing2285@126.com). The data are not publicly available due to privacy.
